# Intramuscular progesterone (Gestone) versus vaginal progesterone suppository (Cyclogest) for luteal phase support in cycles of in vitro fertilization–embryo transfer: patient preference and drug efficacy

**DOI:** 10.1186/s40738-017-0044-y

**Published:** 2017-11-09

**Authors:** Amal Yaseen Zaman, Serdar Coskun, Ahmed Abdullah Alsanie, Khalid Arab Awartani

**Affiliations:** 1 King Faisal Specialist Hospital and Research Center and Taibah University, Zahrawi St، Al Maather, Riyadh, 12713 Saudi Arabia; 2 King Faisal Specialist Hospital and Research Center and Alfaisal University, Zahrawi St، Al Maather, Riyadh, 12713 Saudi Arabia; 3 King Faisal Specialist Hospital and Research Center, Zahrawi St، Al Maather, Riyadh, 12713 Saudi Arabia

**Keywords:** Vaginal, Intramuscular, Progesterone, Luteal phase support, In vitro fertilization, Pregnancy rate

## Abstract

**Background:**

The requirement for luteal phase support (LPS) in stimulated IVF cycles is well established, however drug choice, and route of administration and duration of use are not. This report evaluates patients’ preference and satisfaction by using either vaginal or intramuscular (IM) progesterone (P) supplementation for luteal phase support after in vitro fertilization and embryo transfer (IVF-ET).

**Methods:**

It is a prospective cohort study done in a reproductive and infertility unit in a tertiary care hospital from March 2013 through February 2015 for four hundred and nine patients undergoing IVF-ET. Patients were allowed to choose either vaginal or IM P for LPS. Patient preference and satisfaction, as well as differences in clinical pregnancy rates between the two groups were assessed at one or two time points throughout the study.

**Results:**

There were no statistically significant differences in the patients’ characteristics and clinical outcomes between the two groups. There were 88 pregnancies (38.8%) among patients treated with vaginal p and 62 pregnancies (34%) among IM P patients. Average satisfaction score at the pregnancy test and ultrasound (U/S) visits was similar between both groups.

**Conclusions:**

Patients’ satisfaction and pregnancy rates were similar between vaginal and IM P supplementation.

## Background

The requirement for luteal phase support (LPS) in stimulated IVF cycles is well established, however drug choice, route of administration and duration of use are not [1]. American Society for Reproductive Medicine (ASRM) Position Statement asserts that, “based on available data, progesterone supplementation in IVF cycles yields significantly higher pregnancy rates compared with placebo or no treatment and lower risks for ovarian hyper stimulation syndrome compared with supplementation with human chorionic gonadotropin (hCG)” [2].

A recent Cochrane meta-analysis discussing luteal phase support for ART cycles confirmed that P has a significantly positive effect on clinical pregnancy, live birth and on-going pregnancy rates [3].The pharmacological properties are different according to the route of P administration. It has been reported that IM administration of P results in rapid absorption with higher serum P concentrations [4]. Moreover, IM P has a longer half-life and longer therapeutic levels compared to vaginal route allowing a wider implantation window and less endometrial contractions on the day of the embryo transfer [5]. However availability of IM P has been problematic since it is only available in few countries and injections are considered painful to the most of the patients [6].

An earlier meta-analysis [7] demonstrated no significant difference in treatment outcomes between vaginal and IM P administration. A recent systematic review and meta-analysis also showed that there were no statistically significant differences in live birth rates between IM and vaginal preparations [3]. Different vaginal formulations like gel, insert, ring and suppositories also resulted in similar pregnancy rate and live birth rate [8, 9].

In Saudi Arabia, there is shortage in the IM P supplementation, and a significant number of women were looking for it in spite of reassurance and education been provided that both preparations vaginal and IM P are equal in the effect and outcome. Of well-known that effectiveness is linked to compliance. Patient preference and P efficacy has not previously been studied in Saudi population. The culture in Saudi Arabia has specific traditions that need to be considered when evaluating patients from that region.

The primary objective of this study was to evaluate patients’ preference and satisfaction in both groups when offered the choice of route of progesterone administration. The secondary objective was to evaluate the clinical pregnancy rate between the vaginal and IM P supplementation for LPS after IVF-ET cycles.

## Methods

### Study design

This is a prospective cohort study that included a group of 409 women who underwent IVF-ET treatment at KFSH& RC IVF unit from March 2013 through February 2015. Each woman completed a fresh treatment cycle of IVF-ET, using her own oocytes. On the day of embryo transfer patients were offered to choose between vaginal or IM P for LPS. The study was explained to the patients and they were asked to participate. Patients who agreed to partake in the study were asked to sign an informed consent form and their choice of drug was prescribed. Cyclogest vaginal P used at a dose of 400 mg twice daily (L.D. Collin, Barnstaple, UK) and 50 mg IM Gestone (Ferring Pharmaceutical, Parsippany, NJ, USA).

In both groups, P luteal phase support started on the day of embryo transfer and continued until a negative pregnancy test was confirmed, pregnancy loss occurred, or up to 10 weeks gestation was achieved (ongoing pregnancy). During their second visit for pregnancy test, they were interviewed to fill the satisfaction questionnaire and blood collected for β-hCG. If not pregnant, treatment and study will be terminated after we phone interview the woman. If pregnant, the patient was given a pregnancy ultrasound appointment after 5 weeks according to the IVF Clinic protocol. On their 3rd^.^ visit for pregnancy ultrasound, patients were again interviewed to fill the satisfaction questionnaire for second and last time.

The study was conducted in accordance with the ethical principles of King Faisal Specialist Hospital and Research Center ethical committees. The protocol and its associated Informed Consent Agreement were reviewed and approved by the appropriate Institution committee. Study personnel obtained written informed consent directly from all participants before their entry into the study.

### Inclusion criteria

Healthy women between 18 and 40 years of age, with a body mass index ≤34 kg/m2, a baseline FSH level ≤ 12 mIU/mL on day 3 of menstrual period, a history of infertility or genetic disease requiring IVF or Pre-implantation Genetic Diagnosis (PGD) and a normal uterine cavity were included in the study.

### Treatment protocol

Patients underwent pituitary down-regulation either with GnRH agonist or GnRH antagonist protocol. In GnRH agonist protocol one dose of Leupron depot 3.75 mg IM injection administered during the follicular phase between cycle day (CD) 2 and CD5. Documentation of the down-regulation of the pituitary and ovaries was confirmed by endometrial lining <5 mm and no evidence of ovarian cysts on the transvaginal (TV) U/S. Ovarian cysts were defined as ovarian follicles with a mean diameter ≥ 15 mm). Starting stimulation and following the response depend on TV U/S; estradiol levels were not measured. After documentation of adequate down-regulation of the pituitary/ovarian axis, gonadotropin treatment was started with the use of hMG (Menogon; Ferring Pharmaceuticals, Parsippany, NJ) or recombinant FSH (Gonal F, Merck Pharmaceuticals, Whitehouse Station, NJ, USA). In GnRH antagonist protocol Genarlix (Merck Pharmaceuticals, Whitehouse Station, NJ, USA) 0.5 mg SC started on day 7 of stimulation (fixed antagonist protocol). All women received at least one vial of hMG daily during the period of COH. Ovulation was triggered by administration of 5000 IU or 10,000 IU hCG when three or more follicles had reached a diameter of 18 mm or greater. Oocytes were retrieved 36 h later using single lumen needle without flushing the follicles. Eggs were inseminated either by conventional IVF or by intracytoplasmic sperm injection as appropriate. A maximum of two cleavage stage or two blastocyst stage embryos were transferred on either post retrieval day 3, or days 5, depending on the quality and quantity of embryos available. About 2 weeks after ET, a serum pregnancy test was performed to document biochemical pregnancy. If positive, a repeat serum pregnancy test was performed after 2 days. At about 3 weeks after a positive serum pregnancy test, clinical pregnancy was confirmed by TV U/S. The women who were pregnant continued taking P support until 10 weeks gestational age.

### Assessments

During each visit (i.e., on ET day, on hCG pregnancy test day and on U/S pregnancy confirmation visit at 7 weeks of gestation, some recurrent pregnancy loss patients were scheduled for TV U/S as early as 5 weeks plus, patients were asked by the study investigator to assess their overall health and fill the satisfaction questionnaire. A satisfaction score from 1 to 5 (5 is a high score and 1 is a low score) was used to assess patients’ satisfaction in regard to treatment methods. Responses to the survey assessed compliance and ease of use and other variables such as pain and discomfort associated with the studied drug administration. Assessment of safety and tolerability included documentation of side effects (SE) which is an unpleasant effect of a drug that happens in addition to the main effect (Table 1).

**Table 1:** The questions asked to the patients during their initial, pregnancy test day and U/S day visits

1st Visit (ET day)
AGE
BMI
Endometrial Thickness-
Infertility type
- 1ry
-2ry
Duration of infertility
Menstrual pattern
- regular
- irregular
Infertility factor
-anovulation
-endometriosis
-male factor
-tubal factor
-other
Type of cycle:
IVF
ICSI
IVF/ ICSI
Patient choice:
- Cyclogest
- Gestone
2nd Visit (Preg. test day)
-Pregnancy test:
-ve
+ ve
-Was the type of progesterone changed?
YES
NO
-Side effects:-
Perineal irritation
Rectal itching
Vaginal bleeding
Vaginal or rectal leaking or discharge
Constipation
Discomfort after administration
Hematoma formation
Other
-Patient’s compliance:
-Patients satisfaction: 1(least satisfaction), 2, 3, 4, 5 (most satisfaction)
3rd Visit (U/S day, 7 wks. Preg.)
-Was the type of Progesterone changed?
YES
NO
-Side effects:
Perineal irritation
Rectal itching
Vaginal bleeding
Vaginal or rectal leaking or discharge
Constipation
Discomfort after administration
Hematoma formation
Other
-Patient’s compliance:
-Patients satisfaction: 1(least satisfaction), 2, 3, 4, 5 (most satisfaction)
-Pregnancy outcome:
• Biochemical
• Clinical

### Outcomes

The primary outcome was the percentage of patients preferring IM P as route of administration for LPS during IVF-ET cycles compared to the percentage of vaginal supplementation and their satisfaction with their choice. The secondary efficacy variables were positive β-hCG test rate, clinical pregnancy rate and early spontaneous abortion rate in both groups.

Clinical pregnancy was defined as the presence of one or more gestational sacs detected on ultrasound scan performed 5 weeks after embryo transfer. Biochemical pregnancy loss was defined as a rise in the β-hCG with no evidence of any gestational sac on the ultrasound scan. Miscarriage was defined as a pregnancy loss after U/S confirmation of a positive fetal heart embryo and before 12 weeks gestation.

The expected side effects of IM P i.e. discomfort after administration, hematoma formation and vaginal bleeding) or vaginal P treatment (perineal irritation, inflammation, itching, vaginal or rectal leakage or bleeding), as well as the systemic side effects (e.g. constipation and abdominal pain) were of particular interest.

### Statistics

Comparison of the two groups in terms of demographic and cycle characteristics (age, BMI, duration of infertility and number of IVF cycle) were done by using two independent sample t-test. For categorical values (male factor infertility, positive pregnancy test, pregnancy loss and SEs) we used chi-square test. Mean, median, range and standard deviation (SD) were used as descriptive statistics. Mann-Whitney non-parametric test was used to compare between median score of the two drugs because we are making comparison of two drugs preparation in two occasions (visit). The problem of multiplicity was avoided using Bonferroni correction with a *P*-value to be significant if it falls below 0.025 (0.05/2 = 0.025).

## Results

A total of 409 patients were involved in the study, 227 in the vaginal group and 182 in the IM group; all of them agreed to participate. All were approached either during their routine visits (ET, pregnancy test and U/S day) or through phone interview. 61.2% of the vaginal group completed the ET and pregnancy test visits and the remaining 38.8% became pregnant and all of them completed the whole three follow-up visits (no one lostto followup). Similarly, in the IM group 66% completed two follow-up visits and 34% became pregnant and completed the three visits and none were lost tofollow-up.

(Table 2) shows demographic and cycle characteristics in the vaginal and IM groups. The two groups were without significant differences in age, BMI or cycle characteristics;number of prior cycles and type of stimulation protocol and endometrial thickness on hCG day as well as the duration of infertility was similar for the vaginal and IM P treatment groups (Table 2). Similarly, there was no significant difference between the two groups with regard to treatment outcomes including rates of positive pregnancy tests and pregnancy loss (Table 3). Male factor infertility consider the only infertility diagnosis differing substantially with *P*-value =0.03 (Table 4).

**Table 2:** Demographic features and cycle characteristics

	Vaginal (*n* = 227)	IM (*n* = 182)	*P* value
Age (MEAN and SD)^a^	31.3 ± 4.7	31.6 ± 5	0.5
BMI (MEAN and SD)^a^	28.6 ± 4	27.9 ± 4.3	0.2
Duration of infertility(MEAN and SD)^a^	6 ± 4	6 ± 4	0.35
NO. of cycle (MEAN and SD)^a^	2 ± 1.7	2 ± 1.5	0.84
Long protocol n (%)^b^	152 (67%)	110 (60%)	0.067
GnRh Antagonist n (%)^b^	75 (33%)	72 (40%)	0.3

^a^t- test was used

^b^Chi-square test was used

**Table 3:** Cycle outcome

Cycle outcome	Vaginal (*n* = 227)	IM (*n* = 182)	*P* value
+ve PREG. TEST n (%)^a^	88 (38,8%)	62 (34%)	0.9
Pregnancy loss n (%)^a^	15 (17%)	11 (17,7%)	0.9

^a^Chi-square test was used

**Table 4:** Infertility diagnosis^a^

Primary Cause of inf.	Vaginal (*n* = 227)	IM (*n* = 182)	*P* value
Anovulation n (%)	20 (9%)	13 (7%)	0.5
Male factor n (%)	125 (55%)	119 (65%)	0.03
Pelvic and peritoneal factor n (%)	19 (8.3%)	12 (7%)	0.5
Unexplained n (%)	18 (8%)	17 (9%)	0.6
Other n (%)	45 (19.7%)	21 (12%)	0.02

^a^Chi-square test was used

**Table 5:** Overall side effects for each treatment in pregnancy test and U/S visit^a^

	Vaginal *n* = 227	IM *n* = 182	*P* value
Any side effect n (%)	76 (33.5%)	53 (29%)	0.3
Perineal irritation n (%)	7 (3%)	3 (1.6%)	0.3
Rectal itching n (%)	12 (5%)	1 (0.5%)	0.006
Vaginal bleeding n (%)	10 (4.4%)	4 (2.2%)	0.2
Rectal leak n (%)	22 (9.7%)	0	0.00016
Vaginal leak n (%)	35 (15.4%)	2 (1.1%)	<0.0001
Constipation n (%)	22 (9.7%)	4 (2.2%)	0.002
Discomfort after administration n (%)	3 (1.3%)	33 (18%)	<0.0001
Abdominal pain n (%)	3 (1.3%)	1 (0.5%)	0.4
Diarrhea n (%)	1 (0.4%)	0	0.4
Bloating n (%)	1 (0.4%)	0	0.4
Hematoma formation n (%)	0	3 (1.65%)	0.05

^a^Chi-square test was used

**Table 6:** Patients’ satisfaction in pregnancy test visit and U/S visit

Patients’ satisfaction	Vaginal	IM	*P* value
In preg. Test visit (median)	5	5	0.245
In U/S visit (median)	4.5	5	0.095

There were significant differences between the two groups regarding the side effects. Discomfort after administration was the common side effect reported with IM P. Vaginal leak and rectal leak are the most common SEs with the vaginal group (Table 5). Moreover, vaginal group has significantly more rectal itching and constipation and IM group had three hematoma formation which had borderline significance *P* value = 0.05. Satisfactions were similar and not statistically different between both groups in pregnancy and U/S visits (Table 6).

## Discussion

This analysis provides a, prospective comparison of patient-reported compliance and overall patient satisfaction for vaginal P compared to IM P from a population of women undergoing IVF. The most distinguishing result in this study was the selection of IM route (their own choice) by a good number of patients although they were given information about similar efficacy and outcome for both routes. This might be due to cultural sensitivity regarding use of vaginal route despite pain and discomfort after administration of IM P.

There are limited studies, evaluating administration preferences for different P routes for luteal phase support. One study compared P vaginal inserts (endometrin) to IM P [10], preferences following provider randomization at the start of their luteal phase and at their final visit Patients’ convenience, ease of use lead to higher patient satisfaction with the vaginal route compared to the IM route (82.6% of patients in vaginal group compared with 44.9% in IM group). In our study, patients were given the choice to select their preferred route of progesterone administration on the ET day. 55.5% of patients preferred the vaginal route and the other 44.5% preferred the IM route.

Only a few studies about P for luteal phase support have attempted to assess patients’ treatment satisfaction and ease of use. Previous studies reported better satisfaction with vaginal gel and vaginal insert than IM P [10–12]. Another study assessed the women’s satisfaction with weekly P vaginal ring (VR) versus 8% P gel for luteal phase support after IVF it confirmed that VR was more convenient, less stressful and associated with less leakage than the gel [12]. In our study, patients’ satisfaction with their choice was assessed by using satisfaction questionnaire with scale from one to five (one is least satisfied and five is most satisfied) which showed no significant difference between the groups. Although a significant number of patients had pain and discomfort after administering the IM P it didn’t affect their compliance and satisfaction towards their choice, and all of the patients continued the treatment period with the same drug although they had the option of changing it. Comparing our results to previous studies that confirmed worldwide preference of vaginal route with suppositories, gel or inserts are more commoly desired than the IM route. This suggests that the vaginal route provides an easy-to-use and convenient method without pain and discomfort following administration as compared to the IM route [10, 12–14]. Our study may differ from other published reports due to cultural difference in our population. We believed that cultural differences contributed to our results in our population since vaginal and rectal leakage might interfere with a woman’s daily religious prayers.

Pregnancy outcome including clinical pregnancy and miscarriage rates were assessed in a previous studies and foundno statistically significant differences among both treatment groups [10, 12, 14–19]. Pregnancy outcomes in this study were similar to those reported in previous studies.

Regarding side effects, discomfort after administration has been reported frequently with IM P which is the most common SE reported with this route in all previous studies [10–13] as well asin this study. For the vaginal route, vaginal leakage and rectal leakage are the most common SEs reported during our study. These described undesirable events are expected and specific to each route.

A new route of P administration by aqueous subcutaneous P h shows different pharmacokinetic parameters and lower dosing is needed. This may result in fewer side effects, mainly pain and discomfort after administration. Two large multicenter RCTs demonstrated that the subcutaneous P 25 mg administered once daily is effective and more convenient to the patient [20–22]. It may be suitable for women who prefer to avoid the IM and vaginal routes of administration [20–22]. This drug should be to be tested in Saudi population once it is available.

## Conclusions

Patients’ satisfaction and pregnancy rates were similar between use of vaginal and IMP.

## Abbreviations

ART: Assisted reproductive technology
BMI: Body mass index
CD: Cycle day
E2: Serum estradiol
ET: Embryo transfer U/S Ultrasound
FSH: Follicle-stimulating hormone
GnRH: Gonadotropin-releasing hormone
GnRHa: Gonadotropin-releasing hormone agonist
hCG: Human chorionic gonadotropin
IM: Intramuscular
IVF: In vitro fertilization
LPS: Luteal phase support
OHSS: Ovarian hyperstimulation syndrome
P: Progesterone
PCOS: Polycystic ovarian syndrome
PGD: Preimplantation genetic diagnosis
PIO: Progesterone in oil
Preg: Pregnancy
Pt: Patient
rFSH: Recombinant FSH
SC: Subcutaneously
SD: Standard deviation
SE: Side effect
